# Relationships among morphological variables, cardiovascular fitness during exercise, and thermo-physiological responses under passive heat stress according to Sasang typology

**DOI:** 10.1186/s40101-026-00438-6

**Published:** 2026-08-01

**Authors:** Andrew Gorski, Sang-Hyun Roh, Joo-Young Lee

**Affiliations:** 1 College of Korean Medicine, Kyunghee University, Seoul, Korea; 2 Department of Philosophy, Wonkwang University, Iksan, Korea; 3 Research Institute for Human Ecology, College of Human Ecology, Seoul National University, Seoul, Korea; 4 Department of Fashion and Textiles, College of Human Ecology, Seoul National University, Seoul, Korea; 5 Graphene Research Center for Convergence Technology, Advanced Institute of Convergence Technology, Suwon, Korea

**Keywords:** Sasang constitutional medicine, Physical characteristics, Total sweat rate, Self-identified heat tolerance, Heat waves

## Abstract

**Background:**

The present study explored relationships how Sasang constitutional types were related to morphological characteristics, cardiovascular fitness during exercise, and thermo-physiological responses under passive heat stress.

**Methods:**

Twenty-four healthy males participated in this study. With a questionnaire, the 24 males were classified into the four Sasang constitutional types: 4 Tae-Eum (TE), 5 So-Yang (SY), 15 So-Eum (SE), but 0 Tae-Yang TY) type. Morphological characteristics (height, weight, body surface area, total body fat, and body mass index), cardiovascular fitness during exercise (maximum oxygen consumption [VO2max] and maximum heart rate) and thermos-physiological responses during passive heat stress test (rectal temperature, total sweat rate, thermal sensation, and thermal comfort) were recorded.

**Results:**

The results showed that the TE group had smaller standardized VO2max (ml/kg/min) than the SE and SY group (*P* < 0.05), but unstandardized VO2max (ml/min) showed the opposite tendency (TE > SE). There were differences in thermoregulatory behavior to cold stress between the TE and SY groups (*P* < 0.05). However, no differences among the three constitutional types in rectal temperature, total sweat rate, or subjective perception were found during the passive heat stress. Irrespective of the Sasang types, heavier and fatter subjects showed lower maximum rectal temperature under the passive heat stress (*P* < 0.05).

**Conclusions:**

These results indicated that the Sasang constitutional types correspond to cardiovascular fitness level during exercise rather than thermo-physiological responses during the passive heat stress. Notably, the Sasang constitutional types were primarily reflected in morphological characteristics, rather than in thermo-physiological responses under passive heat stress. Further studies with a sufficient number of subjects including TY individuals during both heat and cold stress tests are required in order to verify the scientific ground of the Sasang typology.

## Introduction

Heat waves during summer are becoming increasingly frequent in the world. Consequently, heat-related disorders have also risen in correlation with these heat waves in Korea [1]. Individual susceptibility or vulnerability to heat stress varies based on several factors, including age, sex, health status, and level of acclimatization. For a considerable number of Koreans, heat-related vulnerability is often understood in terms of “Chejil” (a constitutional type). There are 27,469 traditional Korean medical doctors and 15,124 medical clinics in Korea [2]. Most traditional Korean medicine applies Sasang constitutional theory, which emphasizes integrative and holistic medicine with individual characteristics and personalized medicine approach [3]. Contemporary Sasang constitutional medicine considers a human being as a whole rather than focusing on particular biological systems [3].

Sasang typology is the basis for type-specific prevention, treatment, and rehabilitation procedures using medical herbs and acupuncture combined with appropriate alterations in lifestyle. According to the Sasang typology, humans can be classified into the four constitutional types of Tae-Yang, So-Yang, Tae-Eum, and So-Eum type, which differ in their physiological and psychological features, vulnerability to particular illness, and thermoregulatory responses [3]. It is known that the Big-Little (Tae-So) distinction originated in Confucianism, while the Yin-Yang (Eum-Yang) concept is closely associated with Taoism. The Big-Little distinction not only represents body size but also corresponds to social attitudes (e.g., a Big person represents someone who has more inclusive perspective on society with more fully developed higher cognitive functions). The Yin-Yang corresponds to the rules of nature, embodying the two opposing sides of nature such as introvert-extrovert, cold-hot, wet-dry, moon-sun, night-day, dark-bright, and so on [3]. While there is no exact English translation of the Korean term “Chejil” (a constitutional type), it can be understood as highlighting an individual’s physiological characteristics and innate health traits. However, in practice, Sasang constitutional types refer to the comprehensive physical and mental characteristics of an individual and the treatment paradigms corresponding to these. Also, Sasang types take into account environmental influences and have been the subject of recent and rigorous genetic research [4].

According to Sasang typology, the Tae-Eum type has a higher body mass index (BMI) and bigger chest circumference than to the So-Eum and So-Yang types [5]. Interestingly, Tae-Eum and So-Eum types showed opposite trends in terms of perspiration, temperature preference, and susceptibility to thermal stress. The Tae-Eum type sweats much and feels fresh after sweating, prefers cold to hot weather, usually feels their hands to be warm, and prefers cold drinks over hot drinks, whereas the So-Eum type prefers hot to cold weather, feels their hands to be cold, and prefers hot drinks over cold drinks; these differences can be explained in terms of autonomic reactivity of the sympathetic and parasympathetic nerve systems [6–8]. In particular, So-Eum type females often report cold hands and feet compared to the Tae-Eum females [9]. In traditional Korean medicine, sweat is formed by the combination of *yang qi* with *yin* resulting in perspiration as a form of waste fluid and humor excretion [10].

In modern thermal physiology, however, peripheral skin temperatures and sweating characteristics are associated with the homeostasis of temperature regulation. Peripheral blood vessels are constricted under cold stress, while vasodilation occurs under heat stress. Under cold stress, cold hands and feet would be beneficial for body heat conservation because excessive heat loss from the periphery is prevented, while warm hands and feet are helpful for facilitating heat dissipation to ambient environments. Sweating is a negative thermo-effector feedback responses, which is initiated when body heat content exceeds a certain threshold [11, 12]. Earlier onset and greater sweat volume are beneficial for preventing hyperthermia [11, 12]. Sweating responses can be modified through heat acclimation and acclimatization so that body temperature regulation under heat stress is efficiently achieved [11, 12].

As addressed, the Sasang typology of traditional Korean medicine and modern thermal physiology have different perspectives in body temperature regulation. Sasang constitutional medicine considers heat/cold-related disorders as chronic and integrated symptoms, while thermal physiology considers those disorders as an imbalance between heat production and dissipation. However, the phenomena that the two disciplines are interested in are similar. In this regard, their two perspectives can considered to be similar to some extent, although there are few studies examining this point. Furthermore, with the expectation that global warming will be accelerated due to climate change, there is increasing research interest in increasing human temperature regulation adaptability. By integrating research on Sasang constitutional types and modern thermal physiology, new findings that contribute to enhancing climate adaptability may be achieved. Indeed, very few studies have examined cardiovascular and thermoregulatory responses during active and passive heat stress of the Sasang types. Based on this background, we aimed to examine the temperature regulation responses of the four Sasang constitutional types from thermo-physiological perspectives. The hypotheses were as follows: Firstly, there would be morphological differences among the Sasang constitutional types. Secondly, there would be differences in cardiovascular (aerobic) fitness among the Sasang constitutional types. Thirdly, there would be differences in total sweat rate during passive heat stress among the Sasang constitutional types.

## Methods

### Subjects

**Fig. 1:**
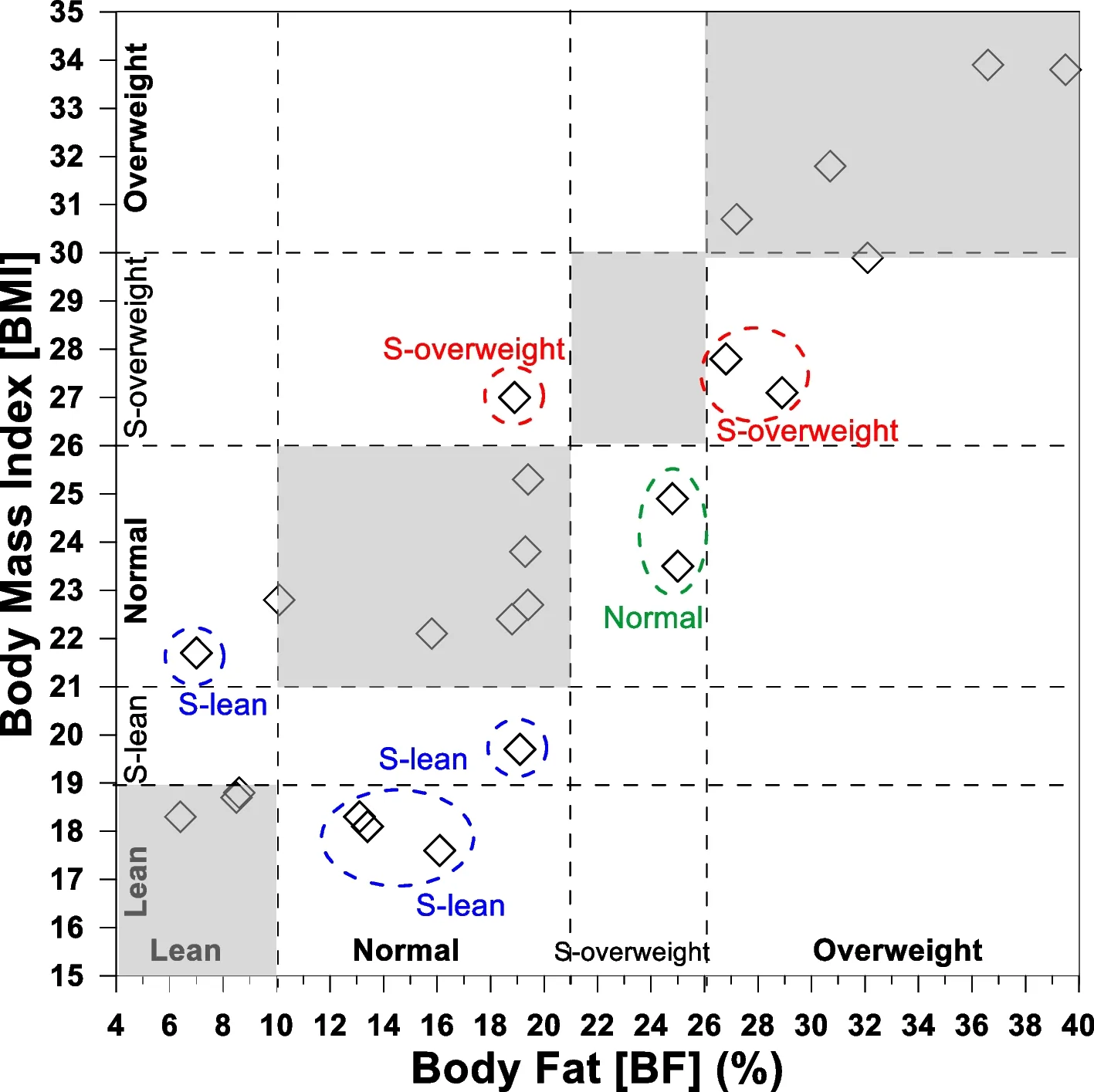
Final body shape classification based on body fat percentage (%) and body mass index (BMI). “S-lean” and “S-overweight” indicate slightly lean and slightly overweight body shapes, respectively. Red, green, and blue circles represent classifications determined by authors based on subjects’ photographs, in conjunction with body fat percentage and BMI

A total of 24 healthy Korean males participated in the present study (Table 1). All volunteers were screened using a health questionnaire and interview to confirm the absence of cardiovascular, metabolic, or other chronic diseases. Only physically active, non-smoking individuals without habitual alcohol consumption were included in the study. These subjects were evenly distributed between underweight and overweight considering total body fat (6.4~39.5%) and body mass index (BMI, 17.6~33.8 kg/m^2^). Total body fat (%) was estimated using a bio-impedance method (InBody 970, InBody Co., Ltd., Seoul, Korea). The body shape of each subject was determined by total body fat (BF%) and body mass index (BMI): Lean BF < 10%, Normal 10 ≤ BF < 21%, Slightly overweight 21 ≤ BF < 26%, Overweight BF ≥ 26%; Lean BMI < 19, Slightly lean 19 ≤ BMI < 21, Normal 21 ≤ BMI < 26, Slightly overweight 26 ≤ BMI < 30, Overweight BMI ≥ 30. The criteria were based on obesity classification standards commonly used for Korean adult males [13]. The 24 subjects were classified as 3 Lean, 5 Slightly lean, 8 Normal, 3 Slightly overweight, and 5 Overweight using the total body fat and BMI criteria above (Table 1, Fig. 1). Body surface area was calculated using the following equation: BSA(cm^2^) = 73.31 × Height (cm)^0.725^ × Weight (kg)^0.425^ [14]. Subjects were instructed to abstain from alcohol and excessive exercise for at least 24 h before participation. All the subjects were informed of the purpose of the study and procedures in advance. Written consent was obtained from all the subjects. The Institutional Review Board of Seoul National University approved this study (IRB No. 2203/002–005).

**Table 1:** Anthropometric characteristics of subjects in the present study

Subject #	Age (y)	Height (cm)	Weight (kg)	Body fat (%)	Body mass index (BMI)	Body shape		
						By body fat [BF] (%)	By body mass index [BMI]	Determined
1	25	171.4	53.9	6.4	18.3	Lean	Lean	Lean
2	25	173.0	65.0	7.0	21.7	Lean	Normal	S-lean*
3	19	173.3	56.3	8.5	18.7	Lean	Lean	Lean
4	20	171.0	55.0	8.6	18.8	Lean	Lean	Lean
5	26	172.0	67.5	10.1	22.8	Normal	Normal	Normal
6	23	171.0	53.5	13.1	18.3	Normal	Lean	S-lean
7	23	178.5	57.7	13.4	18.1	Normal	Lean	S-lean
8	26	181.0	72.3	15.8	22.1	Normal	Normal	Normal
9	24	177.0	55.0	16.1	17.6	Normal	Lean	S-lean
10	22	170.1	64.9	18.8	22.4	Normal	Normal	Normal
11	24	175.0	82.7	18.9	27.0	Normal	S-overweight*	S-overweight
12	26	160.0	50.5	19.1	19.7	Normal	S-lean	S-lean
13	27	184.0	80.7	19.3	23.8	Normal	Normal	Normal
14	24	177.8	71.7	19.4	22.7	Normal	Normal	Normal
15	30	172.0	74.8	19.4	25.3	Normal	Normal	Normal
16	25	173.0	74.5	24.8	24.9	S-overweight	Normal	Normal
17	23	170.0	68.0	25.0	23.5	S-overweight	Normal	Normal
18	27	171.2	81.6	26.8	27.8	Overweight	S-overweight	S-overweight
19	25	187.6	108.1	27.2	30.7	Overweight	Overweight	Overweight
20	25	166.5	75.0	28.9	27.1	Overweight	S-overweight	S-overweight
21	28	178.0	100.7	30.7	31.8	Overweight	Overweight	Overweight
22	25	181.5	98.6	32.1	29.9	Overweight	S-overweight	Overweight
23	26	179.7	109.6	36.6	33.9	Overweight	Overweight	Overweight
24	27	168.5	95.9	39.5	33.8	Overweight	Overweight	Overweight
Mean ± SD	24.8 ± 2.4	174.3 ± 6.0	73.9 ± 17.9	20.2 ± 9.3	24.2 ± 5.1			

*“S-lean” and “S-overweight” represent slightly lean and slightly overweight, respectively

Lean BF < 10%, Normal 10 ≤ BF < 21, Slightly overweight 21 ≤ BF < 26, and Overweight BF ≥ 26; Lean BMI < 19, Slightly lean 19 ≤ BMI < 21, Normal 21 ≤ BMI < 26, Slightly overweight 26 ≤ BMI < 30, and Overweight BMI ≥ 30

### Questionnaire for Sasang constitution classification

We used Questionnaire for Sasang Constitution Classification (QSCCⅡ +, 54 items) to classify Sasang four constitutional types. Although QSCCⅡ + is administered as a questionnaire, Sasang constitutional diagnosis in clinical practice is based on comprehensive physician judgment. The questionnaire was developed to systematize these clinical observations for research applications. Four more questions were added concerning subjects' relation to their thermal environments (Table 2). The additional four items (Questions 55–58) were included to assess self-identified thermal tolerance and behavioral thermoregulation, and were not incorporated into the determination of Sasang constitutional types. The constitutional type that received the most responses was determined to be the respondent’s constitutional type. It is important to note that no person was all one type. Most respondents were roughly half one type with the remaining half being some mix of the other types. From this kind of classification, Korean constitutional medicine treats an individual as So-Yang, Tae-Eum, etc., but tailor diagnosis and treatment more specifically to their particular constitutional makeup.

**Table 2:** Questionnaire to determine the four constitutional types (QSCCⅡ+) and to evaluate behavioral thermoregulation in the present study

#	*QSCCⅡ*+ Question	Responses	Four type*
1	My body physique is …	① Large frame ② Average ③ Small frame	① TY ③ TY, TE
2	My body shape(type) is …	① Large frame ② Average ③ Small frame	① TE ② TY, SY ③ SE
3	The ratio of my upper and lower body is …	① The upper body is larger than the lower body ② The lower body is larger than the upper body ③ My upper and lower body are proportionate in size	① TE ② SE ③ SY
4	My body is…?	① The neck is well-developed, while the waist area is relatively underdeveloped ② The chest area is well-developed, while the hip area is relatively underdeveloped ③ The waist area is well-developed, while the neck area is relatively underdeveloped ④ The hip area is well-developed, while the chest area is relatively underdeveloped	② SY ④ SE
5	My skeleton and body mass is…	① The skeleton is large, and the weight is above average ② The skeleton is small, and the body is leaner than average ③ The skeleton is average, and the body is well-balanced	① TE ② SE ③ SY
6	My chest area is…	① The chest is wide and well-developed (overweight type) ② The chest cavity is very thin and hunched (lean type) ③ The chest is wide and strong (muscular type) ④ The chest is wide and sturdy (sturdy type)	① TE ② SE ③ SY
7	I generally prefer …	① warm food ② cold food ③ no specific preference to food	① SE
8	My hands and feet are	① colder than others ② warmer than others ③ neither colder or warmer than others	① SE ② TE
9	My gait is…	① walks slowly and with dignity ② walks naturally and smoothly (gently) ③ walks briskly (actively) with a slight rocking motion of the torso ④ walks stiffly	① TE ② SE ③ SY
10	I typically …	① tend to sweat a lot and feel refreshed after sweating ② do not sweat much and feel tired after sweating ③ do not feel very tired after sweating	① TE ② SE
11	My personality is…	① honest and decisive ② active and courageous ③ dignified and formal ④ gentle and sincere	① TY ② TE ③ SY ④ SE
12	My working style is …	① I am the type who do not easily give up, no matter what task I am working on ② I am good at starting projects, but I tend to struggle with completing them ③ I prefer working quietly alone rather than moving around and working with others ④ I prefer working in one location and do not like moving around or working outside	① TY ② SY ③ SE
13	I take care of all matters very energetically	① Strongly agreed ② Agreed ③ Not both ④ Disagreed ⑤ Strongly disagreed	① SY ② SY
14	I am very active and have strong will power	① Strongly agreed ② Agreed ③ Not both ④ Disagreed ⑤ Strongly disagreed	① SY, TY ② SY, TE
15	I am not very calculating before I take action	① Strongly agreed ② Agreed ③ Not both ④ Disagreed ⑤ Strongly disagreed	① SE ② SE
16	I can stay in a fixed place for a long time	① Strongly agreed ② Agreed ③ Not both ④ Disagreed ⑤ Strongly disagreed	① TE, SE ② TE, SE
17	I am meticulous and careful	① Strongly agreed ② Agreed ③ Not both ④ Disagreed ⑤ Strongly disagreed	① SE ② SE
18	I restrict my social activities to those with close friends	① Strongly agreed ② Agreed ③ Not both ④ Disagreed ⑤ Strongly disagreed	① SE ② SE
19	I befriend others quite easily, without thinking much about it	① Strongly agreed ② Agreed ③ Not both ④ Disagreed ⑤ Strongly disagreed	① SY ② SY
20	I am more focused on personal matters than social matters	① Strongly agreed ② Agreed ③ Not both ④ Disagreed ⑤ Strongly disagreed	① SE ② SE
21	I think that appearance is more important than internal values	① Strongly agreed ② Agreed ③ Not both ④ Disagreed ⑤ Strongly disagreed	① TY ② TY
22	I cannot befriend others easily because I am reserved in my thoughts	① Strongly agreed ② Agreed ③ Not both ④ Disagreed ⑤ Strongly disagreed	① TE ② TE
23	I am neat and careful	① Strongly agreed ② Agreed ③ Not both ④ Disagreed ⑤ Strongly disagreed	① SE ② SE
24	I am outspoken	① Strongly agreed ② Agreed ③ Not both ④ Disagreed ⑤ Strongly disagreed	① TY, TE ② TY, TE
25	I am very sweet. I can comfort others well	① Strongly agreed ② Agreed ③ Not both ④ Disagreed ⑤ Strongly disagreed	① SE ② SE
26	I am happy to participate in social events	① Strongly agreed ② Agreed ③ Not both ④ Disagreed ⑤ Strongly disagreed	① TE ② TE
27	When I look at someone, I can tell whether or not he/she is diligent	① Strongly agreed ② Agreed ③ Not both ④ Disagreed ⑤ Strongly disagreed	① TY ② TY
28	I am always in a hurry	① Strongly agreed ② Agreed ③ Not both ④ Disagreed ⑤ Strongly disagreed	① SY ② SY
29	I am not comfortable speaking to strangers	① Strongly agreed ② Agreed ③ Not both ④ Disagreed ⑤ Strongly disagreed	① SE ② SE
30	I am timid and always anxious	① Strongly agreed ② Agreed ③ Not both ④ Disagreed ⑤ Strongly disagreed	① SE ② SE
31	I am usually quite sensitive	① Strongly agreed ② Agreed ③ Not both ④ Disagreed ⑤ Strongly disagreed	① SE ② SE
32	I am patient	① Strongly agreed ② Agreed ③ Not both ④ Disagreed ⑤ Strongly disagreed	① TE, SE ② TE, SE
33	I am quite careless	① Strongly agreed ② Agreed ③ Not both ④ Disagreed ⑤ Strongly disagreed	① SY ② SY
34	I am swift and courageous	① Strongly agreed ② Agreed ③ Not both ④ Disagreed ⑤ Strongly disagreed	① TY ② TY
35	I am more masculine than feminine	① Strongly agreed ② Agreed ③ Not both ④ Disagreed ⑤ Strongly disagreed	① SY, TE ② SY, TE
36	I am bothered by small things	① Strongly agreed ② Agreed ③ Not both ④ Disagreed ⑤ Strongly disagreed	① SE ② SE
37	I find it difficult to speak or present in front of others	① Strongly agreed ② Agreed ③ Not both ④ Disagreed ⑤ Strongly disagreed	① SE ② SE
38	I am usually cheerful but have mood swings	① Strongly agreed ② Agreed ③ Not both ④ Disagreed ⑤ Strongly disagreed	① SY ② SY
39	I preferred to be okay even when I am hurt	① Strongly agreed ② Agreed ③ Not both ④ Disagreed ⑤ Strongly disagreed	① SE ② SE
40	When someone looks down on me, I cannot contain my anger	① Strongly agreed ② Agreed ③ Not both ④ Disagreed ⑤ Strongly disagreed	① TY ② TY
41	I do not speak until I am sure that I am right	① Strongly agreed ② Agreed ③ Not both ④ Disagreed ⑤ Strongly disagreed	① SE ② SE
42	I often pretend to know or boast about my knowledge	① Strongly agreed ② Agreed ③ Not both ④ Disagreed ⑤ Strongly disagreed	① SY ② SY
43	I tend to neglect myself but am very polite to others	① Strongly agreed ② Agreed ③ Not both ④ Disagreed ⑤ Strongly disagreed	① SY ② SY
44	I cannot take action in the presence of others	① Strongly agreed ② Agreed ③ Not both ④ Disagreed ⑤ Strongly disagreed	① SE ② SE
45	I am not generous when returning favors to others	① Strongly agreed ② Agreed ③ Not both ④ Disagreed ⑤ Strongly disagreed	① TY ② TY
46	I am often timid and anxious	① Strongly agreed ② Agreed ③ Not both ④ Disagreed ⑤ Strongly disagreed	① SE ② SE
47	I am more feminine than masculine	① Strongly agreed ② Agreed ③ Not both ④ Disagreed ⑤ Strongly disagreed	① SE ② SE
48	I have a desire to achieve more even after I have achieved a considerable goal	① Strongly agreed ② Agreed ③ Not both ④ Disagreed ⑤ Strongly disagreed	① TY ② TY
49	Sometimes I have vomited food or have not been able to swallow food for no specific reason	① Strongly agreed ② Agreed ③ Not both ④ Disagreed ⑤ Strongly disagreed	① TY ② TY
50	When I am not well, sweating makes me feel better	① Strongly agreed ② Agreed ③ Not both ④ Disagreed ⑤ Strongly disagreed	① TY ② TY
51	When I am not well, I have bad digestion	① Strongly agreed ② Agreed ③ Not both ④ Disagreed ⑤ Strongly disagreed	① SE ② SE
52	I am usually very low in energy. I sigh a lot	① Strongly agreed ② Agreed ③ Not both ④ Disagreed ⑤ Strongly disagreed	① SE ② SE
53	I have experienced several episodes of not being, able to move my body, especially my legs, because I felt so low in energy	① Strongly agreed ② Agreed ③ Not both ④ Disagreed ⑤ Strongly disagreed	① SE ② SE
54	I frequently feel uneasy in the chest and stomach after eating	① Strongly agreed ② Agreed ③ Not both ④ Disagreed ⑤ Strongly disagreed	① SE ② SE
	**Questions on behavioral thermoregulation**	**Responses**	
55	Self-identified heat tolerance	① Very vulnerable (intolerable) ② Vulnerable ③ Not both ④ Non-vulnerable ⑤ Very non-vulnerable (tolerable)	
56	Self-identified cold tolerance	① Very vulnerable (intolerable) ② Vulnerable ③ Not both ④ Non-vulnerable ⑤ Very non-vulnerable (tolerable)	
57	Preferred behavior to heat	① Less active than usual ② Less wearing clothes ③ Using air-conditioners or fans ④ Drinking cold water (or cold shower)	
58	Preferred behavior to cold	① More active than usual ② More wearing clothes ③ Using heating systems ④ Drinking hot water (or warm baths)	

*TY Tae-Yang, *TE* Tae-Eum, *SY* So-Yang, *SE* So-Eum

### Experimental conditions and procedure: active and passive heat stress

#### Active heat stress

Subjects visited a laboratory at the same time of day for the maximum oxygen consumption (*V*O_2max_) test. They were instructed to abstain from alcohol and strenuous exercise for 48 h, as well as from food and caffeine for 3 h, prior to their scheduled tests. The *V*O_2max_ test was performed on a motorized treadmill (Aeromill; NihonKohden, Tokyo, Japan) in a climate chamber where air temperature and relative humidity were maintained at 23~24 °C and 40~50%, respectively. The subjects began each trial with a 15-min rest on a chair for stabilization and then warmed up (walking at a speed of 3 km/h without incline) for 5 min. Following the warm-up period, a modified Bruce protocol was initiated followed by a 15-min recovery period on a chair after cessation of exercise. The original Bruce protocol consists of seven stages with progressive increases of speed and incline every 3 min starting from 2.74 km/h at a 10% incline (about 6°), but we reduced the increase in slope for safety (~max. 40%). The subjects wore light clothing (short sleeved shirts and short pants). During the tests, subjects were encouraged to perform as well as they could. However, the tests were terminated when they reached volitional fatigue. Each test was retrospectively examined and confirmed as a true maximal test when two of following four criteria were met: (1) plateau or increase in oxygen uptake ≤ 2.1 mL/kg/min with an increased work load; (2) respiratory exchange ratio (RER) ≥ 1.10; (3) HR ≥ 95% of age-predicted HR_max_ (220—age in years); and (4) ratings of perceived exertion (RPE) ≥ 18. Maximal oxygen consumption was expressed both as an absolute value (*V*O_2max_, ml·min⁻^1^; non-standardized) and as a body-mass–normalized value (*V*O_2max_, ml·kg⁻^1^·min⁻^1^; standardized).

#### Passive heat stress

The climate chamber was set at an air temperature of 30 °C with 50% relative humidity. The experiments were conducted between March and April in Korea, which is Spring. When subjects arrived, they took a rest and put on prepared underpants and short pants in a pre-room with a neutral air temperature (*T*_a_ of 23~25 °C). After drinking 300 ml of water to assure hydration, rectal temperature sensors were equipped by the subjects. To estimate total sweat rate, subjects were weighed three times each on a scale, during the trial, as well as pre- and post- heat stress trial. The passive heat stress test consisted of a 10-min rest on a chair followed by a 60-min leg immersion in a 42 °C water. Subjects immersed their calves and feet into the water. Subjects completed a questionnaire after the passive heat stress test during recovery in a thermal-neutral room.

### Measurements

#### Active heat stress

Before and after each trial, subjects’ seminude body weight was measured on a calibrated scale with 1-g resolution (ID2; Mettler-Toledo, Albstadt, Germany). In all trials, gas exchange variables such as oxygen uptake (*V*O_2_), carbon dioxide production (*V*CO_2_), ventilation (VE), and respiratory exchange ratio (RER) were continuously measured at a sampling interval of 10 s throughout the rest, exercise, and recovery periods using an automatic respirometer (Quark CPET, Italy). Prior to each trial, the respirometer was calibrated using room air and standard gas mixture (5.03% CO_2_, 15.00% O_2_, balance nitrogen); calibration was done using a 2 L syringe. Heart rate (HR) was recorded every second using an HR monitor (RS400; Polar Electro, Finland). Gas exchange and HR data during the last 30 s of exercise period were averaged and considered the maximal values. Subjects were also asked to provide their ratings of perceived exertion (RPE; Borg’s 6–20 scale) every 2 min.

#### Passive heat stress

To estimate total sweat rate, subjects were weighed on a calibrated scale (Resolution 1 g; ID2, Mettler-Toledo, Germany) before and after each trial. Total body mass loss did not consider respiratory water loss separately. Rectal temperature (*T*_re_) was measured every 5 s using a data logger (LT-8A, Gram Corporation, Japan) and a thermistor sensor, which was inserted nearly 16 cm beyond the anal sphincter. The following subjective perceptions were recorded at the 55th min during leg immersion: 9-point thermal sensation (4: very hot, 3: hot, 2: warm, 1: slightly warm, 0: neutral, −1: slightly cool, −2: cool, −3: cold, −4: very cold), and 7-point thermal comfort (3: very comfortable, 2: comfortable, 1: a little comfortable, 0: neither, −1: a little uncomfortable, −2: uncomfortable, −3: very uncomfortable).

### Data analyses

Statistical analysis was performed using SPSS 26.0. One-way ANOVA and Duncan’s post hoc test were undertaken to test significant differences in parametric data among the three Sasang groups (TE, SY, and SE groups). A Kruskal–Wallis test was conducted to test significant differences in non-parametric data among the three Sasang groups. Pearson’s or Spearman’s correlation coefficients were calculated to test relationships between morphological variables and thermo-physiological responses. Body shapes were scored with “1 Lean, 2 Slightly lean, 3 Normal, 4 Slightly overweight, to 5 Overweight” for the correlation analysis. Significance was set at *P* < 0.05. All data were presented as mean ± SD.

## Results

### Four constitutional types

**Fig. 2:**
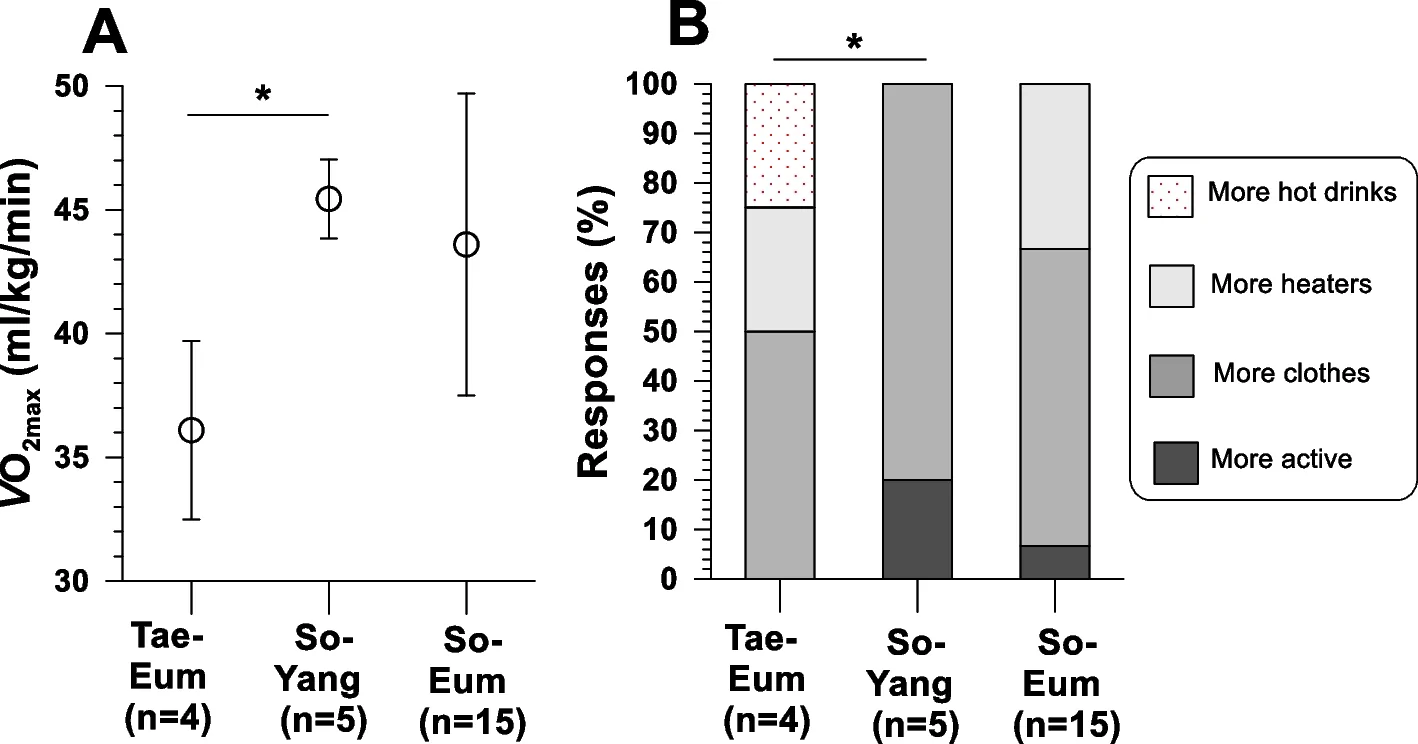
Three constitutional types and maximal oxygen consumption (*V*O_2max_) (**A**) and preferred thermoregulatory behavior to cold (**B**) (*P < 0.05). One-way ANOVA and Duncan’s post hoc test were undertaken to test significant differences in *V*O_2max_ among the three Sasang groups (**A**), while a Kruskal–Wallis test was conducted to test significant differences in response rate(%) among the three Sasang groups (**B**)

Among the 24 subjects, there were 4 Tae-Eum, 5 So-Yang, and 15 So-Eum, with no subjects classified as Tae-Yang (Table 3). The Tae-Eum group had greater body weight and body surface area than the other two groups (Table 4). Although no statistically significant group difference was observed, Tae-Eum individuals generally exhibited lower surface area–to–body mass ratios compared with the other Sasang types, with substantial overlap in individual values across groups (Table 4). BMI was greater for the Tae-Eum group when compared to So-Eum, but the difference was not significant (Table 4, *P* = 0.087). Classification by total body fat (%) was similar to those by BMI even though there was no statistical significance. Most Tae-Eum individuals assessed themselves as being overweight, while other two groups assessed themselves as being normal (Table 4). During the *V*O_2max_ test, the Tae-Eum group (34.9 ± 3.1 ml/kg/min) had a significantly smaller value compared to the So-Yang group (45.4 ± 1.6 ml/kg/min) (*P* = 0.041) (Fig. 2A). However, non-standardized *V*O_2max_ was greater for the Tae-Eum than for the So-Eum group (3419 ± 812 ml/min for TE vs. 2926 ± 566 ml/min for SE, *P* = 0.144; Table 4). In terms of behavioral temperature regulation in response to cold, the So-Yang group preferred wearing additional clothing and more activities (motions), whereas the Tae-Eum group did not prefer being more active (*P* = 0.044) (Fig. 2B). There were no significant differences in average and maximum rectal temperature and total sweat rate during the passive heat stress test among the three Sasang groups. The Tae-Eum group felt hotter and more uncomfortable during the passive heat stress test compared to the So-Yang group, but the group differences were not significant (Table 4).

**Table 3:** Number of responses to the questions which characterized four constitutional types (unit: N)

Subject #	Four constitutional types				Determined type
	Tae-Yang	Tae-Eum	So-Yang	So-Eum	
1	3	3	4	**22**	So-Eum
2	8	7	**9**	**9**	So-Yang*
3	4	6	4	**18**	So-Eum
4	5	6	6	**15**	So-Eum
5	2	5	7	**16**	So-Eum
6	6	4	3	**10**	So-Eum
7	6	8	7	**12**	So-Eum
8	5	**13**	6	10	Tae-Eum
9	3	3	4	**16**	So-Eum
10	3	4	6	**10**	So-Eum
11	8	8	**12**	8	So-Yang
12	3	4	5	**14**	So-Eum
13	5	4	**11**	10	So-Yang
14	5	5	7	**18**	So-Eum
15	4	9	**11**	8	So-Yang
16	2	2	8	**19**	So-Eum
17	8	7	**10**	7	So-Yang
18	4	10	5	**13**	So-Eum
19	2	**10**	6	1	Tae-Eum
20	3	6	3	**14**	So-Eum
21	2	9	8	**12**	So-Eum
22	5	**11**	8	7	Tae-Eum
23	1	12	9	**16**	So-Eum
24	3	**15**	5	6	Tae-Eum

*So-Yang and So-Eum responses are the same, but the subject was classified as “So-Yang” due to Tae-Yang response is high

**Table 4:** Morphological and physiological responses according to three constitutional types

Variables	Three constitutional types			*F*-value	*P-*value
	Tae-Eum (*N* = 4)	So-Yang (*N* = 5)	So-Eum (*N* = 15)		
Morphological and physical characteristics					
Height (cm)	179 ± 8	174 ± 5	172 ± 5	1.202	0.118
Body weight (kg)	**93 ± 13**	**74 ± 8**	**68 ± 18**	**2.040**	**0.035**
Body surface area, BSA* (m^2^)	**2.17 ± 0.18**	**1.93 ± 0.12**	**1.84 ± 0.22**	**3.102**	**0.023**
BSA/body weight (cm^2^/kg)	234 ± 25	261 ± 13	276 ± 34	1.840	0.057
Total body fat (%)	28.7 ± 9.9	17.9 ± 6.6	18.8 ± 9.1	1.124	0.136
Body mass index (BMI)	29.1 ± 5.0^a^	24.3 ± 2.0^ab^	22.9 ± 5.2^b^	1.581	0.087
*V*O_2max_ (ml/min)	3,419 ± 812	3,472 ± 210	2,926 ± 566	1.133	0.144
*V*O_2max_ (ml/kg/min)	**36.1 ± 3.6**^**b**^	**45.4 ± 1.6**^**a**^	**43.6 ± 6.1**^**a**^	**3.887**	**0.041**
HR_max_ (bpm)	198 ± 8	202 ± 13	195 ± 10	0.690	0.476
Passive heat stress test					
Total sweat rate (g/trial)	275 ± 56	281 ± 84	249 ± 59	1.122	0.553
Maximum *T*_re_ (^o^C)	37.5 ± 0.2	37.5 ± 0.3	37.7 ± 0.4	1.199	0.356
Thermal sensation (4 very hot, 3 hot, 2 warm, 1 slightly warm, 0 neutral, −1 slightly cool, −2 cool, −3 cold, −4 very cold)	3.1 ± 0.5	2.3 ± 0.8	2.7 ± 0.9	1.079	0.260
Thermal comfort (3 very comfortable, 2 comfortable, 1 a little comfortable, 0 neither, −1 a little uncomfortable, −2 uncomfortable, −3 very uncomfortable)	−1.9 ± 0.5	−0.9 ± 1.1	−1.3 ± 1.1	1.030	0.455
Questionnaire					
Body shape (1 Lean, 2 Slightly lean, 3 Normal, 4 Slightly overweight, 5 Overweight)	4.3 ± 1.0	2.8 ± 1.1	2.7 ± 1.3	1.888	0.115
Self-identified heat tolerance (1 Very vulnerable, 2 Vulnerable, 3 Not both, 4 Non-vulnerable, 5 Very non-vulnerable)	2.5 ± 1.0	3.2 ± 0.8	2.6 ± 1.0	1.566	0.405
Self-identified cold tolerance (1 Very vulnerable, 2 Vulnerable, 3 Not both, 4 Non-vulnerable, 5 Very non-vulnerable)	2.5 ± 1.3	3.2 ± 0.8	2.5 ± 1.2	0.665	0.458

*BSA(cm^2^) = 73.31 × Height(cm)^0.725^ × Weight(kg)^0.425^ [14]

One-way ANOVA and Duncan’s Post-hoc test were undertaken to test significant differences in parametric data among the three Sasang groups (TE, SY, and SE groups). A Kruskal–Wallis test was conducted to test significant differences in non-parametric data among the three Sasang groups

^a, b, ab^indicate statistically homogeneous groups identifi ed by the post hoc test

### Relationships between anthropometric factors and thermoregulatory variables

We analyzed the correlations between anthropometric and physical factors (age, height, weight, total body fat, BMI, body shape, *V*O_2max_, and HR_max_) and thermoregulatory variables (increase in rectal temperature, maximum rectal temperature, total sweat rate, thermal sensation, and thermal comfort). There were no significant correlations between the factors anthropometric-physical factors of age, height, *V*O_2max_, or HR_max_ and thermoregulatory parameters during the passive heat exposure. No significant relationship between total sweat rate and total body fat or BMI was found even though they were positively correlated (*r* = 0.195, *P* = 0.361 for total body fat; *r* = 0.342, *P* = 0.102 for BMI). However, body shape was significantly related with various parameters, such that individuals, who cognized themselves as being more overweight, had greater total body fat and BMI as well as smaller *V*O_2max_ and lower maximum rectal temperature during the passive heat stress (Table 5).

**Table 5:** Relationships between morphological and physiological factors and three constitutional types

		Morphological and physical factors			Passive heat stress test			
		Total body fat (%)	BMI	VO_2max_ (ml/kg/min)	Total sweat rate	T_re___max_	Thermal sensation	Thermal comfort
Body shape*	Pearson’s r	0.895	0.927	−0.590	0.258	−0.465	−0.222	−0.204
	*P*	< 0.001	< 0.001	0.003	0.224	0.022	0.297	0.339

* 1 Lean, 2 Slightly lean, 3 Normal, 4 Slightly overweight, 5 Overweight

**Fig. 3:**
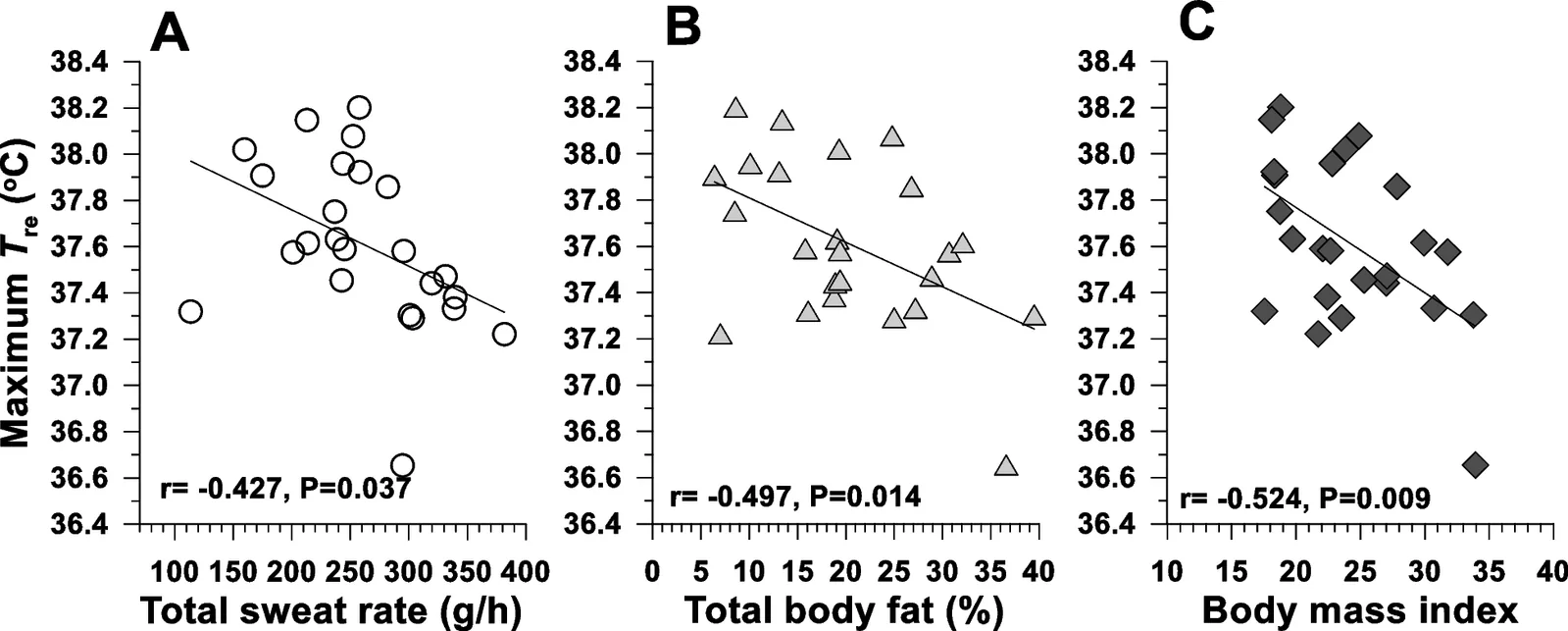
Relationships between total sweat rate (**A**), total body fat (**B**), and body mass index (**C**) and maximal rectal temperature during a 42 °C-leg-immersion for 60 min in a hot environment

Maximum rectal temperature was significantly related to body weight, total body fat, BMI, body shape, and total sweat rate (all *P*s < 0.05, Fig. 3). Specifically, greater total sweat rate, total body fat (%), and BMI were associated with lower maximum rectal temperature during the passive heat exposure (Fig. 3, *P* < 0.05). Maximum rectal temperature also was not significantly related to the thermal sensation or thermal comfort. Anthropometric characteristics did not show significant correlation with total sweat rate, thermal sensation, or thermal comfort during the passive heat stress test. Total sweat rate did not show any significant correlation with thermal sensation or thermal comfort during the passive heat stress test.

### Self-identified heat and cold tolerance

Self-identified heat and cold tolerances were correlated with various variables. Older individuals among the 24 subjects (19 ~ 30 years old) identified themselves as being less vulnerable to heat (*r* = −0.480, *P* = 0.017). Those who identified themselves as being more vulnerable to heat also reported greater thermal discomfort when exposed to the passive heat (*r* = 0.490, *P* = 0.015). In addition, lean individuals were more likely to identify themselves as being more vulnerable to cold (*r* = 0.424, *P* = 0.039). The So-Yang group identified themselves as less vulnerable to heat and cold than the other two types, but these differences were not significant (Table 4).

## Discussion

This study is original in that it explored the differences between the Sasang constitutional types of Korean medicine from a cardiovascular and thermo-physiological perspective during active and passive heat stress tests. Furthermore, this research is significant in that we recruited young males with a wide range of body shapes (160.0~187.6 cm in height, 53.9~109.6 kg in body weight, 6.4~39.5%BF, and 17.6~33.9 in BMI) in order to explore the relationships between anthropometric factors and thermoregulatory parameters according to Sasang types. Of the three hypotheses addressed in the introduction, the first hypothesis was partially accepted because the Tae-Eum group had greater BSA and smaller body surface area to body mass ratio than other groups (*P* < 0.05). The second hypothesis “there would be differences in cardiovascular fitness (*V*O_2max_) among the Sasang constitutional types” was accepted. The Tae-Eum group showed greater *V*O_2max_ (ml/kg/min) than that of the So-Eum group, but non-standardized *V*O_2max_ (ml/min) showed the opposite result. The third hypothesis was rejected because there were no significant differences in thermo-physiological parameters, such as maximum rectal temperature, total sweat rate, thermal sensation, and thermal comfort during the passive heat stress, among the three Sasang constitutional groups. A more detailed discussion on morphological factors, cardiovascular fitness during exercise, and thermo-physiological responses during passive heat stress are as follows.

### Sasang constitutional types and body morphology

In Korean medicine, the questionnaire (QSCCⅡ+) is understood as a research-oriented proxy for clinician-based Sasang diagnosis rather than a measure of subjective self-perception. In Sasang constitutional theory, an individual with a larger physique and skeleton, more body weight, and a more developed upper body compared to lower body is considered to have characteristics of Tae-Eum [15]. On the other hand, an individual with a smaller physique, less body weight, and more developed lower body than the upper body is seen as having So-Eum characteristics [15]. The Tae-Eum type has abdominal obesity compared to other types [16, 17]. In general, BMI and total body fat are greater for the Tae-Eum type than Se-Eum [3]. The surface area to body mass ratio of the Tae-Eum type appeared to be lower, implying lower heat dissipation efficiency [15]. Because of this, it seems that the Tae-Eum type is more vulnerable to heat stress than other types [15].

The results of the present study showed that the Tae-Eum group (*n* = 4) had a greater body weight, BSA, and BMI when compared to the So-Eum group (*n* = 15). These indicate consistency between the results obtained through self-reported questionnaires (QSCCⅡ+) and actual measurements using a body scale, bioelectrical impedance method, etc. The measured height, body weight, and total body fat of subjects who assessed themselves to have a larger physique were indeed greater and bigger than the So-Eum or So-Yang groups. Classifying the Sasang constitutional types based on the self-reported questionnaire is not purely circular reasoning (i.e., “the big subject is called Tae-Eum because ‘Tae’ means big”), because the questionnaire (QSCCⅡ+) consists of various anthropometric aspects such as body physique, diet, sweating, activities, tastes, preferences, personalities, etc. We acknowledge that QSCCⅡ+ places substantial weight on morphological characteristics. The strong contribution of physique to Sasang classification is not unexpected and aligns with the conceptual framework of the present study, which aims to reinterpret Sasang typology from a physiological anthropological perspective. The present results reflect the phenotype-oriented nature of Sasang typology as a historically established human classification system. We do not seek to validate Sasang typology as an independent diagnostic system, but to examine how a morphology-oriented classification corresponds to cardiovascular fitness and thermo-physiological responses. It is interesting that surface area to body mass ratio was smaller for the Tae-Eum type than the So-Yang or So-Eum type (*P* = 0.057), because the small value means that Tae-Eum type’s body shape is not efficient to dissipate body heat under heat stress.

### Sasang constitutional types and cardiovascular fitness during graded exercise

An interesting result of the present study was the difference in *V*O_2max_ between the three groups. *V*O_2max_ (ml/min) tended to be greater for the Tae-Eum group than that of So-Yang and So-Eum groups, indicating that the Tae-Eum individuals had the greatest cardiovascular capacity among the three groups, but the difference was not statistically significant (*P* = 0.144). Rather, standardized *V*O_2max_ (ml/kg/min) was significantly smaller for the Tae-Eum group than that of So-Yang and So-Eum groups. Pham and colleagues [15] compared oxygen consumption of 304 subjects during a maximal exercise test, and found that the Tae-Eum type had lower *V*O_2max_ (ml/kg/min) than other types. Park and colleagues [18] measured resting metabolic rate (RMR) of 395 healthy subjects for 15 min and reported that the Tae-Eum type had significantly higher RMR (kcal/day) but lower standardized RMR (kcal/kg/day) than other types. The Tae-Eum type tended to have a lower heat production at rest [15]. Shim and colleague [5] hypothesized that the lung system of the Tae-Eum type was weak, indicating lower metabolic energy production. Kim and colleague [19] found that Tae-Eum type had a significantly lower ATP-linked oxygen consumption rate than the other types based on whole-exome sequencing analysis.

Questions 52 and 53 of the Sasang constitution questionnaire are related to energy metabolism and indicate that the So-Eum type has the lowest metabolic energy level. This may seem to contradict the present *V*O_2max_ (ml/kg/min) findings, but is consistent with the *V*O_2max_ (ml/min) findings that the So-Eum group had a smaller aerobic capacity than the Tae-Eum group. In this regard, it is important to focus on the unit of maximum oxygen consumption (*V*O_2max_). In exercise physiology, *V*O_2max_ is typically expressed per unit of body weight to assess energy efficiency, whereas IUSP [20] defines *V*O_2max_ as the maximum rate at which an organism can take up oxygen in the following unit: “ml · min^–1^” or “l · min^–1^.” The Sasang constitution questions #52 and #53 connote energy expenditure at the individual level (of an organism). When looking at the value of *V*O_2max_ of an individual without standardizing body weight, the So-Eum group showed a lower value compared to the Tae-Eum and the So-Yang groups due to their smaller body weight and BMI. However, the present results based on the standardized *V*O_2max_ verify that cardiovascular fitness is less efficient for the Tae-Eum group than other types.

### Sasang constitutional types and sweat rate under passive heat stress

In Korean Sasang constitutional medical theory, cold and heat (“Han-Yeol” 寒熱) are very important concepts. “Han (寒)” refers to energy with a cold nature. Individuals with an excess of Han have relatively cold bodies, cold hands and feet; they are vulnerable to cold. “Hanzeung (寒症)” refers to a condition where cold energy accumulates in the body, which is believed to be more common in the So-Eum type. On the other hand, “Yeol (熱)” represents energy with a warm or hot nature, indicating an excess of internal energy or an overly active state of energy. Individuals with an excess of “Yeol” feel hot easily, have warm hands and feet, sweat easily, experience facial flushing, or feel thirsty. “Yeoljeung (熱症)” refers to a condition caused by the accumulation of heat in the body, which is believed to be more common in Tae-Eum type.

Hypothermia and hyperthermia of thermal physiology are caused by an imbalance between heat production and heat dissipation. The concept clearly differs from Han-Yeol symptoms in traditional Korean medicine. Indeed, the concept of “cold/hot nature” and “cold/hot energy” in the Han-Yeol theory is something not addressed in modern physiology. For modern physiologists, the concept of “cold energy” sounds like oxymoron (e.g., living dead) because thermal energy in physics is related to the molecular movement of object. However, if we understand Hanjeung and Yeoljeung not as separate conditions but as the result of a disruption in the balance of Han and Yeol, there might be some overlap between the Han-Yeol theory and modern thermal physiology. Remember that hypothermia or hyperthermia occurs due to imbalance between heat production and heat dissipation.

In the questionnaire (QSCCⅡ+), Question #8 (temperature of the hands and feet), Question #10 (the amount of sweating and mood after sweating), and Question #50 (mental sweating and refreshing) are related to Han-Yeol. Warm hands and feet, excessive sweating, and feeling refreshed after sweating correspond to the nature of Tae-Eum, whereas relatively cold hands and feet, insufficient sweating, and fatigue after sweating correspond to the nature of So-Eum. We assumed that the Tae-Eum group would show a greater sweat rate when compared to the So-Eum group during the passive heat stress. The total sweat rate was 275± 56 g/trial for the Tae-Eum group and 249 ± 59 g/trial for the So-Eum group without any significance (*P* = 0.553, Table 4). Although the third hypothesis was not accepted due to the statistical insignificance, it needs to be addressed that the present study was conducted under passive heat stress. Different results in total sweat rate might be obtained during vigorous exercise in a hot environment (i.e., active heat stress). Due to the large body size and great muscle mass of Tae-Eum individuals, metabolic energy during exercise would be higher for Tae-Eum compared to So-Eum, which could lead to faster sweating onset and a greater sweat rate.

Sasang constitutional medicine considers faster sweating onset and a greater sweat rate as indicating susceptibility to heat stress, and the Tae-Eum type is more susceptible to heat compared to the So-Eum type [15]. However, the concept of “susceptibility” should be distinguished from vulnerability because the early onset and great amount of sweating is considered as beneficial responses to facilitate body heat dissipation and alleviate body heat strain under heat stress.

### Relationships between morphological and physiological parameters under heat stress

To better understand the physiological mechanisms underlying the group-level findings described above, additional analyses were conducted to examine the relationships between morphological characteristics and physiological responses under passive heat stress. In the present study, during passive heat stress greater BMI, greater total body fat, and greater sweat rate were significantly associated with lower maximum rectal temperature (Fig. 3). It is understandable that greater sweat rate mitigated rectal temperature increase. Because the linking between the larger body size and greater sweat rate was not significant in the present study, interpretation based on morphology is necessary. For instance, larger individuals receive less heat per unit of body weight and body surface area during the passive heat stress (limited amount of hot water). The leg-immersion test involves external heat coming from the hot water without increasing metabolic heat from muscles. Larger individuals possess higher heat capacity (e.g., it takes more heat to boil 1 L of water when compared to 0.5 L of water). Therefore, under constant passive heat stress, smaller individuals could be more vulnerable to hyperthermia when compared to larger individuals because of their lower heat capacity.

### Limitations and suggestions

Among the 24 subjects in the present study, there were no Tae-Yang individual, and the numbers of Tae-Eum and So-Yang were relatively smaller compared to So-Eum. Typically, Tae-Yang were the least represented, with only 7 individuals (0.6%), compared to 335 So-Eum (30.2%), 530 Tae-Eum (47.8%), and 237 So-Yang (21.4%) [21] Hahm et al. [22] and Jang et al. [23] found no Tae-Yang among the 142 and 2,528 patients, respectively. Park et al. [18] found 2% Tae-Yang, 23% So-Yang, 65% Tae-Eum, and 9% So-Eum among the 107 male subjects. Lee et al. found less than 0.1% Tae-Yang type [3]. Although Tae-Yang is known to be the least common among Koreans, similar numbers from all the four constitutional groups should be recruited for a more balanced comparison in the future.

Secondly, the present results on the thermo-regulatory responses cannot be extrapolated to responses under active heat stress, as we measured core body temperature and total sweat rate during a passive heat stress test. Thermoregulatory responses during exercise reflect metabolic heat production from major muscles and might be more strongly influenced by body physique and body mass. The present results during the passive heat stress did not involve metabolic rate that increased by exercising muscles. The amount of muscles affects body shape and body mass. According to Riesenfeld [24], when the rats were exposed to extreme cold and heat, it appeared that large bodied rats survived longer in cold and small-bodied rats survived longer in heat.

Thirdly, since no cold stress test was performed in the present study, relationships between the four constitutional characteristics and thermo-physiological parameters were not fully explored. Given that larger body size and higher total body fat (%) reduce cold strain from cold stress, it is necessary to investigate physiological responses under cold stress conditions. Further studies should conduct cold stress tests as well as active heat stress tests involving exercise, to comprehensively understand relationships between the Sasang constitutional types in traditional Korean medicine, anthropometric factors, and thermo-physiological responses under heat and cold stress.

## Conclusions

This study experimentally examined whether cardiovascular fitness and thermophysiological responses to passive heat stress reflect Sasang constitutional types. The results demonstrated that Sasang typology was closely associated with morphological characteristics and cardiovascular fitness. Tae-Eum individuals exhibited greater body weight, body surface area, and body mass index, as well as higher absolute *V*O_2max_ but lower body-mass–normalized *V*O_2max_ and a smaller surface area–to–body mass ratio compared with So-Eum individuals, suggesting lower aerobic efficiency in the Tae-Eum type. In contrast, no significant differences were observed among Sasang types in thermoregulatory parameters, including rectal temperature and total sweat rate, during passive heat stress. These findings indicate that Sasang typology is more strongly reflected in body morphology and cardiovascular fitness than in thermophysiological responses under passive heat stress. Future studies with larger and more balanced samples, including Tae-Yang individuals, are required to further validate these relationships.

## Data Availability

The datasets used and/or analyzed during the current study are available from the corresponding author on reasonable request.
